# Improved isolation of murine hepatocytes for *in vitro *malaria liver stage studies

**DOI:** 10.1186/1475-2875-6-169

**Published:** 2007-12-20

**Authors:** Lígia A Gonçalves, Ana M Vigário, Carlos Penha-Gonçalves

**Affiliations:** 1 Instituto Gulbenkian de Ciência, Rua da Quinta Grande, 6, 2781-901 Oeiras, Portugal

## Abstract

**Background:**

Primary hepatocyte cultures are a valuable tool for the understanding of cellular and molecular phenomena occurring during malaria liver stage. This paper describes an improved perfusion/dissociation procedure to isolate hepatocytes from mouse liver that is suitable for malaria studies and allows reproducible preparation of primary hepatocytes with consistent cell yields and controlled purity.

**Results:**

This protocol is a detailed description of a technique to isolate and culture mouse hepatocytes and represents an improvement over previous descriptions of hepatocyte isolation for malaria studies, regarding three technical aspects: (1) dissociation reagents choice; (2) cell separation gradient and (3) cell purity control. Cell dissociation was optimized for a specific collagenase digestion media. The cell dissociation step was improved by using a three-layer discontinuous gradient. A cell purity check was introduced to monitor the expression of CD95 on hepatocytes using flow cytometry methods.

**Conclusion:**

The procedure described allows reproducible recovery of one to three million hepatocytes per preparation with cell purity of about 90% as determined by FACS analysis. Completion of the protocol is usually achieved in about four hours per preparation and pooling is suggested for multiple preparations of larger number of cells.

## Background

Hepatocytes represent the vast majority of liver cells and are responsible for the main specific hepatic functions, such as carbohydrate transformation, bile production and protein production and storage. In the course of malaria infection, hepatocytes are a critical site for malaria parasite to establish themselves in the mammalian host. Within hepatocytes, individual parasites occupy a single vacuole, in which it grows rapidly and produces the first generation of red blood cell-infectious merozoite forms. The intrahepatic stage takes, approximately 42 h in rodents and 5–10 days in human malaria, allowing for extensive cellular and molecular interactions between parasite and host [1-4]. To study these exo-erythrocytic stages of malaria parasites, several *in vitro *model systems have been developed using human and rodent hepatocyte cell lines. Immortalized cells are easy to manipulate, but have obvious limitations as they show significantly divergent behaviour as compared to primary hepatocyte cultures and *in vivo *infection [5]. Moreover, it is expected that hepatocyte infection by *Plasmodium *differs depending on the host genetic background [6]. In this context, primary hepatocytes are a valuable tool for the understanding of cellular and molecular phenomena occurring during malaria liver stage. This protocol describes an efficient and reproducible procedure based on perfusion/dissociation procedure to isolate and culture primary hepatocytes for malaria liver stage studies.

The saga of hepatocyte isolation began 40 years ago with a steadily improvement of preparation methods including applications of the technique to malaria parasite studies [7-9]. The method for preparation of intact hepatocytes was first described in 1969 [10] and since then has undergone many modifications. The most commonly used technique was introduced in 1976 and relies on a two-step procedure, including *in situ *perfusion and purification based on cell density [11]. Perfusion uses Ca^2+ ^free medium, critical for cell separation, and subsequently Ca^2+ ^rich medium containing Ca^2+ ^dependent collagenase, for digestion. Appropriate collagenase treatment is absolutely crucial for hepatocyte preparation. Frequently, the difficulties in obtaining good cell dissociation are attributed to crude collagenase contamination with other proteases, which varies depending on the batch. Incidentally, some of these contaminant proteases appear to be required for good cell isolation, but their identity is still uncertain [12]. Another critical step is hepatocyte enrichment. Almost all existing purification protocols for murine primary hepatocytes rely on centrifugation through a discontinuous 60% (1.076 g/ml) Percoll gradient [13]. This is intended to remove non-parenchymal cells, death cells and cell debris, but the degree of purity of hepatocyte preparations has been difficult to access.

Here, a complete description of this technique is provided and introduced a number of improvements over published techniques allowing reproducible hepatocyte isolation, consistent cell yields and quantification of final hepatocyte enrichment. The optimization started by the testing of several collagenase batches and modified the protocol for a commercially available collagenase medium (Hepatocyte Liver Digest Medium from Gibco). After, different Percoll densities configurations were tested until was accomplished superior hepatocyte separation, using a three-layer discontinuous Percoll gradient [14]. In addition, a hepatocyte purity control by flow cytometry analysis was introduced. Mouse hepatocytes prepared by this method are suitable for *Plasmodium *infection as well as immunohistochemistry, gene expression and gene functional studies on monolayer primary cultures.

## Results

### Perfusion apparatus setup

The perfusion system uses a peristaltic pump with adjustable speed. The system is setup by mounting the peristaltic pump with long silicone tubing immersed in a water bath. The water bath temperature is adjusted so that the perfusion fluid is at 37°C at the tube outlet. The pump speed must be set at constant flow of 6 ml/min. To prepare the liver lobes perfusion is necessary to attach an Abocat (18 G and 30 mm length) to the tube outlet end and fill the tubing with Hepatocyte Liver Perfusion Medium (1x) (Gibco).

### Reagent setup

The method uses different reagents that should be prepared in advance.

#### Percoll solutions

Three different Percoll density solutions are used (1.12 g/ml, 1.08 g/ml and 1.06 g/ml). For 1.12 g/ml solution we used 2.63 ml of Percoll, 0.3 ml of PBS (10x) and 64.6 μl of water; for 1.08 g/ml solution we used 2.86 ml of Percoll, 0.5 ml of PBS (10x) and for 1.63 ml of water and for 1.06 g/ml solution we used 2.085 ml of Percoll, 0.5 ml of PBS (10x) and 2.42 ml of water. These solutions must be kept at room temperature.

#### Cell culture media

Two different media were prepared for the primary hepatocyte cultures: Williams' Complete medium (WCM), that was prepared by adding to Williams' Medium E (1x) with GlutaMAX I (Gibco) 4% of Foetal Calf Serum (Gibco) and 1% Penicillin/Streptomycin (Gibco); and Williams' Supplemented medium (WSM), that was prepared by adding to Williams' Medium E (1x) with GlutaMAX I (Gibco) 4% of Foetal Calf Serum (Gibco), 1% Penicillin/Streptomycin (Gibco), 50 ng/ml of Epidermal Growth Factor (BD), 1 μg/ml Insulin (Sigma), 10 μg/ml of Transferrin (Sigma), and 1.3 μg/ml of Hydrocortisone (BD).

#### Plate coating

A 0.2% solution of gelatine was prepared by mixing 1 g of gelatine (Merck) in 500 ml of PBS (1x), dissolved by heating above 60°C and filter-sterilized. This solution was used to coat 24 well plates (200 mm^2^/well) with 400 μl per well. The plates were let open and air drying for about 30 min in a laminar flow cabinet, then the liquid excess was rejected, avoiding scratching the gelatine surface and the plate was air dried for additional 30 min.

### Liver dissection

**Figure 1:**
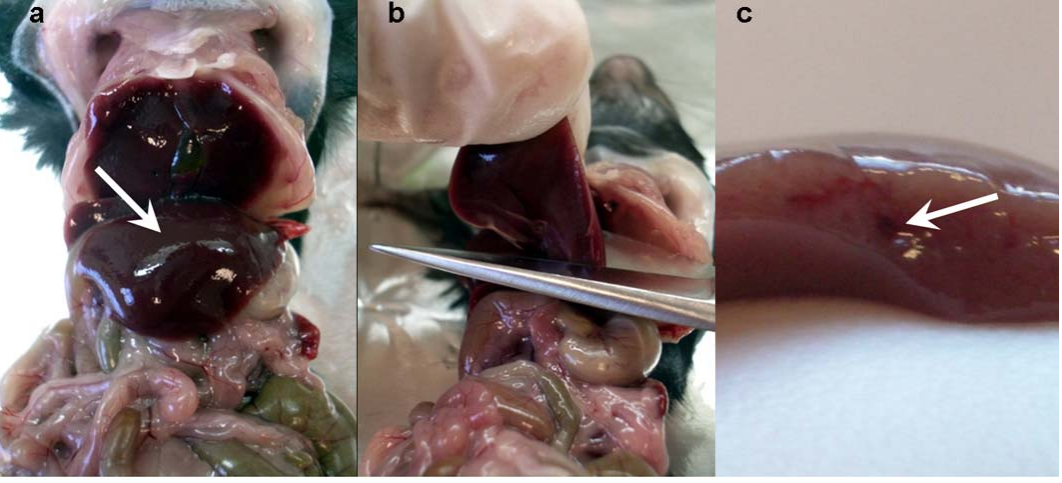
Liver cutting. (a) Left lobe, indicated by arrow. (b) Excision of liver lobe with a single cut, exposing the main blood vessel as shown in (c).

The hepatocytes were isolated from adult mouse livers (8–14 week of age). All animal procedures were in accordance with applicable regulations on animal experimentation and welfare. This procedure was approved by the Animal Care and Use Committee, at the Instituto Gulbenkian de Ciência. The liver lobe is dissected by opening the peritoneal cavity and using surgery scissors (straight tip and cutting edge around 40 mm long, appropriate for soft tissues) to cut the left liver lobe. The cut of the liver lobe should be done with a single cut and in such a manner that the main blood vessel of the lobe is exposed, as shown in Figure 1. The liver lobe was then collected into a 15 ml Falcon tube containing 5 ml of Williams Medium E (1x) with GlutaMAX I (Gibco).

### Liver perfusion

After liver dissection, the liver lobe was transferred onto a piece of gauze placed on the hand. The tip of an Abocat needle (18 G and 30 mm length) was introduced into the main blood vessel and the perfusion was started using Hepatocyte Liver Perfusion Medium (1x) (Gibco). The perfusion was kept until the liver lobe is drained off blood and appears pale, at this stage the Hepatocyte Liver Perfusion Medium was changed to Hepatocyte Liver Digest Medium (1x) (Gibco). It is useful, at this stage, to let an air bubble enter the tubing as this helps to keep the two media apart inside the tubing. Perfusion with Hepatocyte Liver Digest Medium (1x) (Gibco) was kept until the liver lobe felt very soft. This is a critical step, as this medium contains collagenase, and excessive digestion should be avoid to prevent cell death (Table 1). Normally, 30 ml of Hepatocyte Liver Digest Medium were enough for proper digestion.

**Table 1:** Frequent causes unsuccessful hepatocyte isolation.

**Step**	**Problem**	**Possible Reason**	**Solution**
Perfusion	Poor cell dissociation	Poor digestion	Make sure the Digestion solution is at 37°C
		Tissue disruption	Lower down perfusion speed
	Cell death	Excessive collagenase digestion	Reduce the time/volume of perfusion with Hepatocyte Liver Digest Medium
Cell isolation	Low recovery	To many cells loaded on the gradient	If using more than three lobes, increase the number of gradient tubes
		Gradient disturbance	Setup new gradient, making sure the layers do not mix
		Altered gradient densities	Check that the gradient centrifugation temperature is kept at 20°C and Percoll solutions at room temperature

### Cell dissociation

Subsequently to liver digestion, the perfused liver lobe was transferred to a Petri dish containing 10 ml of Hepatocyte Liver Perfusion Medium. Then, with the help of two 1 ml pipettes the liver capsule disrupted. At this stage it is possible to check the perfusion success as upon liver capsule disruption, a fine cloud of cells should spread in the medium as a sign of successful dissociation. Poor cell dissociation usually can be improved by perfusion optimization (Table 1).

The cell suspension was forced through a 100 μm Cell Strainer (BD) into a 50 ml Falcon tube. The tube was filled up to 30 ml with Hepatocyte Liver Perfusion Medium, and centrifuged at 135 g for 30 seconds. At this point, one can choose either to ressuspend the pellet in 24 ml of WCM and move on to the fractionation step or to ressuspend the cells in 3 ml of WCM and store them at 4°C, while continuing to perfuse another mouse liver lobe. Dissociated cells from successive liver digestions can be pooled up to a final volume of 24 ml.

### Hepatocyte isolation

For each 24 ml suspension of dissociated cells, is necessary to mount four Percoll gradient tubes. Using the previously prepared Percoll solutions, 3 ml of 1.12 g/ml solution was applied on the bottom of a polystyrene conical tube (30 ml Universal, Sterilin), over which 5 ml of 1.08 g/ml solution was carefully laid. Finally, 5 ml of 1.06 g/ml solution was laid. Handling of the prepared gradients should avoid mixing the density layers.

**Figure 2:**
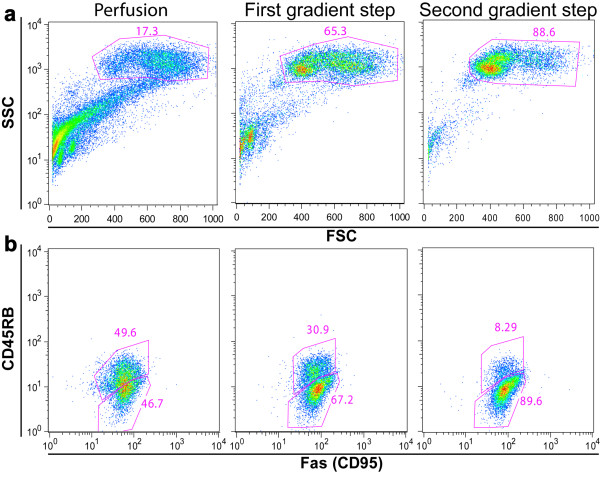
Checking hepatocyte enrichment by flow cytometry analysis. (a) Enrichment of cells within the hepatocyte gate and loss of cell debris along the hepatocyte isolation procedure (perfusion, first and second gradient step). (b) The enriched hepatocyte preparation of the second gradient step is also enriched for cells not expressing CD45RB and expressing CD95 at low levels.

Cell suspension was applied over the Percoll gradient by carefully laying 6 ml of cell suspension in each gradient tube, avoiding gradient disturbance (Table 1). The cell suspension was fractionated by centrifugation at 750 g during 20 min, without brake, at 20°C (Percoll solutions should not be centrifuged below 20°C). After Percoll gradient centrifugation the two upper layers that contain cell debris and non-parenchymal cells were carefully pipetted out and discarded. Then, the lowest layer that contains the live hepatocytes was collected from the four Percoll gradient tubes and dispensed in two 50 ml Falcon tubes previously containing 10 ml of WCM. The Falcon tubes were filled up to 40 ml of volume of WCM before centrifugation at 355 g (20°C) during 10 min. Subsequently, the cell pellets from the two Falcon tubes were ressuspend and pooled in a final volume of 6 ml of WCM. For the second round of cell isolation, an additional Percoll gradient tube was prepared. This second gradient step significantly increases cell purity (Figure 2). Similarly to the first round, the cell suspension was applied over the Percoll gradient, and centrifuge at 750 g during 20 min, without brake. The two upper layers were discarded and the lowest cell layer was collected in a 15 ml Falcon tube. To wash the cells the tube was filled up with WCM, centrifuged at 355 g (4°C) 10 min and the pellet was ressuspend in 15 ml of WCM. The washing was repeated and the pellet was ressuspend in 1 ml of WCM.

### Purity control

To evaluate the hepatocyte enrichment of these preparations we used FACS analysis (Figure 2) and stained cells at different steps of the hepatocyte isolation procedure with anti-CD95 PE, CD45RB-biotin and streptavidin-APC from BD-Pharmingen, The hepatocyte morphological gate was defined on basis of size and confirmed by the presence of high frequency of CD95 low cells. In fact, CD95 is expressed at low levels by hepatocytes and its expression is not detectable in other cell types present in the liver. To detect the presence of other contaminating large cells within the hepatocyte gate we used the CD45RB monocytes/macrophage lineage marker.

### Hepatocyte plating

Viable hepatocytes were counted on a haemocytometer, using trypan blue staining. Depending on cell yield and experiment design 5 × 10^4 ^to 1 × 10^5 ^hepatocytes were used in each culture well in a volume of 700 μl of WCM.

The cell suspension was distributed in the pre-coated 24 well plates, the cells were let to adhere for 4 h in the incubator (37°C and 5% CO_2_) and then changed to WSM.

### Viability assessment

**Figure 3:**
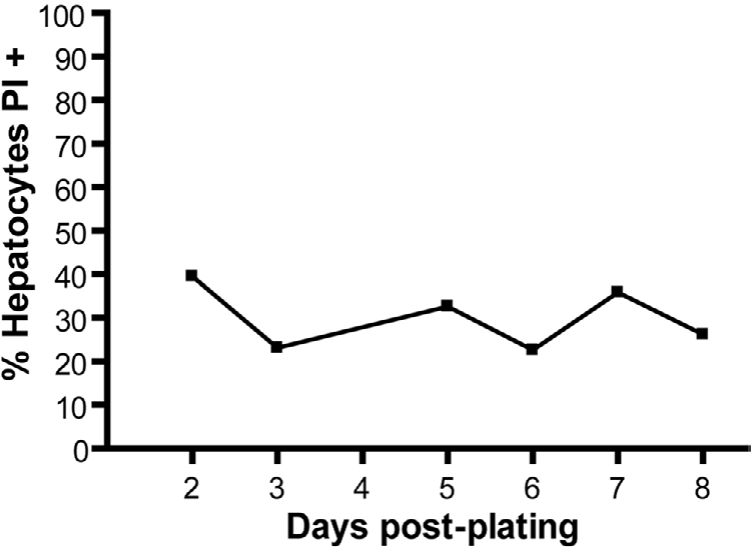
Cells culture viability. Time-course analysis of cell survival in hepatocyte cultures. Dead cells were measured as positive cells to propidium iodide staining in flow cytometry analysis.

Viability of isolate hepatocytes estimated by trypan blue staining is usually 80–90%. Viability of hepatocyte cultures was also monitored during 8 days in culture by FACS analysis using propidium iodide staining. Briefly, cells in culture were detached by standard Trypsin/EDTA treatment, centrifuged, suspended in PBS and stained with propidium iodide 10 μg/ml. Percent of propidium iodide positive cells were detected in the FL3 channel of a FACScalibur instrument (Becton & Dickinson). It is likely that the detachment procedure induces some cell death, but the results indicate that the hepatocyte culture maintains stable viability during at least 8 days (Figure 3).

### Development of *Plasmodium *exo-erythrocytic forms

**Figure 4:**
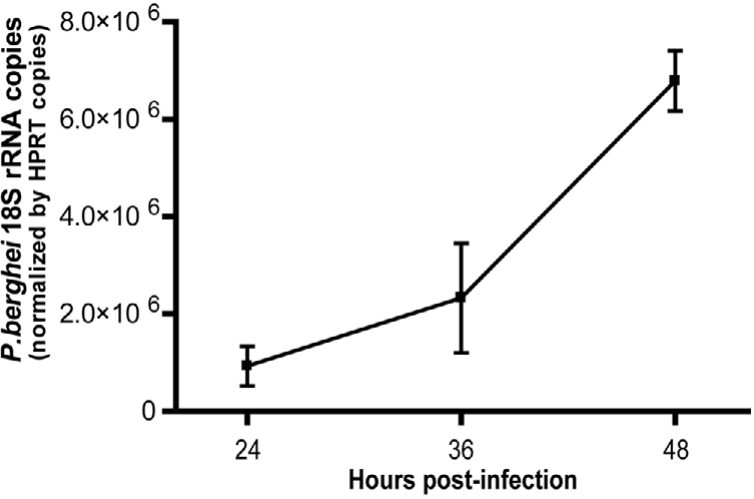
Plasmodium quantification in hepatocyte culture. *P. berghei *18S rRNA was quantified by real-time PCR in cultured hepatocytes of C57Bl/6 mice at the indicated times after infection with 3 × 10^4 ^sporozoites at 24 h post-plating.

**Figure 5:**
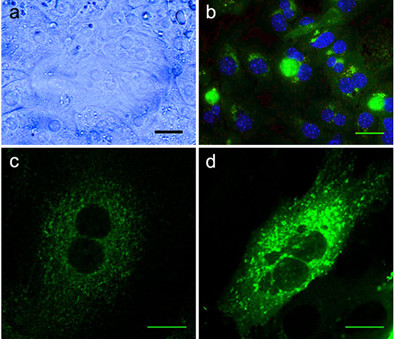
Cell biology applications of cultured hepatocytes. (a) Light microscopy image of a hepatocyte monolayer at 24 h culture. (b) Immunofluorescence image of hepatocytes 36 h after infection with GFP-expressing *Plasmodium berghei *and DAPI staining. (c) Confocal image of hepatocyte stained with monoclonal antibodies to Transferrin receptor labelled with Alexa488 (Invitrogen). d) Confocal image of hepatocyte transfected with an EGFP construct using Lipofectamine reagent (Invitrogen). Bars, 20 μm.

To show that the hepatocyte cultures sustain the development of parasite liver stages, a time-course experiment was performed that quantified the parasite growth in sporozoites-infected cultures (Figure 4). *Plasmodium berghei *ANKA sporozoites were obtained from dissection of salivary glands from infected female *Anopheles stephensi *mosquitoes. Sporozoites suspensions in RPMI medium added at concentration of 3 × 10^4 ^sporozoites per culture well. Parasite rRNA was quantified by real-time PCR. Briefly, cells were collected at indicated times, with the help of a cell scraper, immediately homogenized in denaturing solution (4M guanidine thiocyanate, 25 mM sodium citrate pH 7.0, 0.5% sarcosyl and 0.7 % β-mercaptoethanol in DEPC treated water) and total RNA was obtained using RNeasy Mini Kit (Qiagen). One microgram of total RNA was converted to cDNA (Transcriptor First Strand cDNA Synthesis Kit, Roche) and cDNA specific to *P. berghei *18S rRNA was amplified with primers NYU-Pb1 5'-AAG CAT TAA ATA AAG CGA ATA CAT CCT TAC-3' and NYU-Pb2 5'-GGA GAT TGG TTT TGA CGT TTA TGT-3'. The real-time PCR reactions were performed in ABI Prism 7900HT system using ABI Power SYBR Green PCR Master Mix. Absolute *P. berghei *18S rRNA estimates were normalized for mRNA of Hypoxanthine Guanine Phosphoribosyl-transferase (HPRT), a mouse housekeeping gene. Parasite liver stages development is also detectable by fluorescent microscopy of hepatocytes infected with sporozoites from green fluorescent protein (GFP)-transgenic *P. berghei *ANKA (Figure 5b).

### Immunofluorescence labeling and transfection of hepatocytes

Cell transfection was performed with Lipofectamine (Invitrogen) or Fugene6 (Roche) according to manufacture guidelines. Briefly, 0.5 mg of an EGFP-tagged DNA vector (Invitrogen) was mixed with 2.5 μl of Lipofectamine and 200 μl of Optimem (Invitrogen) or 1 mg of an EGFP-tagged DNA vector was mixed with 5 μl of Fugene6 and 200 μl of Optimem, mixtures were allowed to rest for 15 minutes at room temperature and added to cells. Cells were left in transfection mixture for three hours at 37°C before the medium was changed to normal growth medium. Cells were left to express the EGFP tagged DNA for 24 hours before cell fixation (Figure 5d).

For immunofluorescence labeling, cells were fixed in 4% paraformaldehyde (Sigma) in PBS. Fixed cells were permeabilized and blocked with 0.05% saponin (Sigma) 1% foetal calf serum (Invitrogen) and 1% bovine serum albumin (Sigma), for 30 minutes at 37°C. Adequate primary antibodies were incubated in the same solution as above and left for one hour. All secondary antibodies were obtained from Invitrogen and used at a dilution of 1:400 in the same solution as above (Figure 5c). Cells were mounted on MOWIOL (Calbiochem). Images were acquired using a Leica SP5 confocal microscope.

## Discussion

The hepatocyte enrichment here described is obtained by two successive gradients entail a net cell loss. This procedure usually allows recovery of about 1–3 × 10^6 ^cells per liver lobe. However, the recovery yield may vary significantly, depending on the mouse strain. Routinely, were obtain cell preparations containing 90% of hepatocytes, and cell debris was about 10% or less of the detected events according to flow cytometry criteria (Figure 2). After labelling with CD45RB and Fas (CD95), hepatocytes can be identified in flow cytometry as cells having high side scatter, low expression of CD45RB and constitutively expressing CD95 [15,16]. In fact, these cell preparations are amenable for flow cytometry analysis, either on freshly isolated cells or after culture (standard Trypsin/EDTA treatment can be used to detach cultured cells).

**Figure 6:**
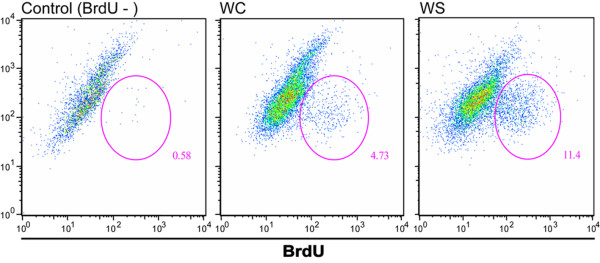
Hepatocyte proliferation. Flow cytometry analysis of hepatocyte proliferation, measured by BrdU incorporation after 72 hours of continuous labelling. The plots show considerable increase in cell proliferation when the cells are cultured in Williams supplemented medium (WSM) as compared to Williams complete (WCM).

Cells for infection with malaria liver stage sporozoites should be kept at least 24 h in culture in WCM. This allows the formation of a stable cell monolayer (Figures 5a and 5b), if enough cells are put initially in culture. When the goal is to infect at 24 h post-plating it is advisable to plate no less than 1 × 10^5 ^cells in a 200 mm^2 ^well (e.g. 24 well plates). To infect after 48 h post-plating, is advisable to start with less cells (5 × 10^4^) and maintain the cells in WSM until 12 h before infection, after which the medium must be changed to WCM, allowing the cells to proliferate (Figure 6) and achieve confluence on a monolayer. Hepatocytes cultured in WSM are stable for about one week (Figure 3), after which they start to die and loose typical characteristics. These hepatocyte cultures sustain *Plasmodium *liver forms development that is detectable both by molecular methods (Figure 4) and microscopy examination (Figure 5b).

The cultured cells when attached resist well to manipulation, allowing immunohistochemistry procedures and transfection assays (Figures 5c and 5d). The experience shown that these cells are better transfected when using Lipofectamine rather Fugene (20% vs. 6% transfection rate). After culture, it is also feasible to collect cells with the help of a cell scraper and RNA lysis solution, and extract RNA for hepatocyte and parasite gene expression analysis.

Although hepatocytes prepared with this technique can be successful used for a range of biological applications it is difficult to directly compare it with the performance of other preparation methods where relevant amounts of other cell types may distort estimations of cell viability, cell counting and cell proliferation.

## Authors' contributions

LAG conceived the protocol, designed and performed the experiments, drafted the manuscript. AMV gave assistance to LAG. CPG participated in study design and coordination and drafted the manuscript. All authors read and approved the final manuscript.
